# Pathways of Intergenerational Risk: Examining the Association Between Maternal Adverse Childhood Experiences and Child Socio-Emotional and Behavioral Concerns at 8 Years of Age

**DOI:** 10.1177/10775595241279365

**Published:** 2024-08-28

**Authors:** Jenney Zhu, Nicole Racine, Suzanne Tough, Sheri Madigan

**Affiliations:** 1 2129 University of Calgary, Calgary, AB, Canada; 2 Alberta Children’s Hospital Research Institute, Calgary, AB, Canada; 3 Department of Psychology, 6363 University of Ottawa, Ottawa, ON, Canada; 4 Children’s Hospital of Eastern Ontario Research Institute, Ottawa, ON, Canada

**Keywords:** adverse childhood experiences, child and adolescent development, families

## Abstract

Support has been found for the intergenerational transmission of risk from maternal adverse childhood experiences (ACEs) to child outcomes. Less research has focused on longitudinal psychosocial pathways that account for this transmission. In the current study, path analysis examined mediating pathways (i.e., maternal adult attachment insecurity, romantic relationship functioning, and maternal anxiety and depression symptoms) in the association between maternal ACEs and internalizing and externalizing concerns among their child at eight years of age. Participants included 1,994 mother-child dyads from a prospective longitudinal cohort sample. Maternal ACEs were significantly associated directly with child internalizing concerns (β = .06, *p* = .025) and indirectly via both maternal attachment anxiety and avoidance, lower romantic relationship functioning, and depression, (β = .002, *p* = .006; β = .003, *p* = .005, respectively). Maternal ACEs were directly associated with child externalizing concerns (β = .06, *p* = .018) and indirectly via both maternal attachment anxiety and avoidance, lower romantic relationship functioning, and depression, (β = .001, *p* = .008; β = .002, *p* = .010, respectively). This study identified several maternal risk factors that have implications for downstream internalizing and externalizing concerns among their children.

## Introduction

The ubiquity of adverse childhood experiences (ACEs), which includes abuse, neglect, and household dysfunction, in the general population has been highlighted by a recent meta-analysis of 206 studies showing that approximately 60% of individuals in population samples experienced at least one ACE, while 16% of individuals experience four or more ACEs (Madigan et al., 2023). The importance of strengthening the understanding of the consequences of ACEs is highlighted by the finding that ACEs and negative health outcomes typically operate in a dose-response manner. That is, the greater number of ACEs experienced, the greater the likelihood of problematic physical, mental, and relational outcomes (Felitti et al., 1998; Hughes et al., 2019; Petruccelli et al., 2019; Zhu et al., 2023). More recently, research efforts have extended to advancing the understanding of whether a parent’s history of ACEs prior to the age of 18 have later implications for their child’s developmental and mental health outcomes. Research has shown that parent ACEs have been associated with greater mental health concerns among their children, including internalizing and externalizing problems (Racine et al., 2023). However, far less research has been conducted on the pathways that account for this association.

### Psychosocial Mechanisms of Transmission Between Caregiver ACEs and Child Psychopathology

Theoretically, transmission from caregiver ACEs to child mental health may be understood using the Family Stress Model, which suggests that life stressors may cause distress and strain in family relationships, negatively impacting parenting and ultimately leading to diminished health and well-being among children (Conger et al., 2002; Masarik & Conger, 2017). A recent meta-analysis examined mechanisms of transmission from maternal ACEs to child outcomes and the authors concluded that more research is needed to map out specific pathways for how maternal ACEs exert their influence on child outcomes (Ma et al., 2022). To date, several intermediary pathways have been proposed, including maternal attachment insecurity, romantic relationship functioning, and anxiety and depression symptoms, but have yet to be examined together, and thus require further empirical testing.

#### Adult Attachment Insecurity

Attachment theory suggests that interactions with caregivers help to form our mental representations of relationships and these attachment styles allow individuals to form expectations in subsequent relationships. For example, children who experienced sensitive and responsive caregiving, including contingent responsiveness to child cues and signals, tended to develop secure attachment, including confidence that their caregiver could be relied upon and would be available when needed (Madigan et al., 2024). In contrast, children who had caregivers who inconsistently met, or consistently failed to meet, their needs may develop insecure-anxious attachment or insecure-avoidant attachment, respectively (Madigan et al., 2024). Anxiously attached individuals were likely to seek closeness, fear rejection, and overestimate threats from romantic partners in adulthood. In contrast, individuals with avoidant attachment were likely to behave in ways that attempt to deny their attachment needs by avoiding closeness, dependence, and intimacy with relationship partners (Ainsworth et al., 1978; Bowlby, 1979; Mikulincer et al., 2003).

Given that ACEs include experiences of abuse, neglect, and maltreatment in the context of a relationship with a caregiver, it is reasonable to expect that parent history of exposure to ACEs may increase the risk for insecure attachment styles in adulthood, including anxious and avoidant attachment. Indeed, adult attachment avoidance and anxiety have both been associated with past exposure to child adversity (e.g., Bifulco et al., 2006; Corcoran & McNulty, 2018). Cowan and colleagues (2019) demonstrated that insecure attachment among mothers predicted anxious and harsh parenting. There is support for the role of attachment insecurity as a pathway that accounts for the association between maternal ACEs and child outcomes. Specifically, a meta-analysis by Ma and colleagues (2022) demonstrated that maternal attachment insecurity was a significant mediator of the association between maternal ACEs and early emotional and behavioral concerns, including externalizing concerns and anxiety/withdrawal among their children. As such, maternal adult attachment insecurity, specifically anxious and avoidant attachment, is likely to mediate the association between maternal ACEs and child internalizing and externalizing concerns.

#### Relationship Functioning

Maternal romantic relationship functioning may have important implications for the association between maternal ACEs and child internalizing and externalizing concerns. Past research has found that higher self-reported relationship quality between caregivers was positively associated with greater caregiver-child engagement (Carlson et al., 2011). In contrast, conflict between co-parents has been associated with poorer child adjustment (Martin et al., 2017). Existing research has demonstrated that ACEs were associated with decreased romantic relationship satisfaction and even abusive relationship experiences, such as intimate partner violence (Eyisoylu & Erdem, 2023; Zhu et al., 2023). Subsequently, past research has demonstrated that children exposed to intimate partner violence, which itself represents an ACE, tended to have poorer outcomes, including greater mental health concerns, including anxiety and behavioral problems, and lower cognitive functioning (Gartland et al., 2021). Taken together, maternal romantic relationship functioning is likely one pathway that explains the association between maternal ACEs and child internalizing and externalizing concerns.

Romantic relationship functioning between parents may impact child well-being via family systems theory, which suggests that all relationships between members of a family are interconnected. As such, relationships between parents may impact relationships between parent and child (Browne et al., 2015). One of the mechanisms in which parental relationship functioning may impact the parent-child relationship is via parental mental health.

#### Maternal Mental Health

Past research has demonstrated that romantic relationship functioning was positively associated with maternal mental health (Braithwaite & Holt-Lunstad, 2017). Moreover, existing research has highlighted the role of maternal mental health concerns following ACE exposure, which may have implications for child outcomes (Cooke et al., 2019). ACEs may increase risk for mental health concerns via biological, psychological, and social pathways (Sheffler et al., 2020). For example, exposure to ACEs can lead to increased allostatic load. That is, exposure to stress leads to activation of the sympathetic nervous system, parasympathetic nervous system, and the hypothalamic-pituitary-adrenal axis, which represent adaptive responses to stressors. However, repeated activation of these circuits, as is often the case with ACEs, can lead to wear and tear on the body’s adaptive circuits (Berg et al., 2017). With respect to the association between maternal mental health and child internalizing and externalizing concerns, ACEs may increase susceptibility to stress and negatively impact the resources needed to cope with stress, leading to greater mental health difficulties. Further, maternal mental health concerns may have downstream consequences for mothers’ interactions with their children. For example, Racine and colleagues (2021) demonstrated meta-analytically that maternal ACEs were associated with both pre- and post-natal depression. A meta-analysis of 193 studies conducted by Goodman and colleagues (2011) found significant associations between maternal depression and child outcomes, including higher externalizing and internalizing concerns and general psychopathology. Thus, it is plausible that maternal mental health would mediate the association between maternal ACEs and child internalizing and externalizing concerns.

### Gaps in Existing Literature

A scoping review by Zhang et al. (2023) on the association between parent ACEs and child mental health outcomes revealed several notable gaps in the literature to date. First, among the 35 studies included in the scoping review, over 50% examined child outcomes between birth and five years, and thus, they concluded there is a need for additional research across the developmental spectrum of childhood. Second, most studies to date have examined single mediators, but developmental psychopathology theory would suggest that there are likely to be cascading effects of multiple psychosocial risk factors (i.e., pathways of risk; Cicchetti, 1993) that account for the association between maternal ACEs and child mental health problems, including internalizing (e.g., depression, anxiety) and externalizing (e.g., aggression, conduct problems) concerns. Third, most studies have examined parental mental health and parenting behaviors (e.g., sensitivity) as potential mediators; however, there is a paucity of research focusing on how family level variables, such as maternal adult attachment and romantic relationship functioning, may serve as intermediatory factors in the pathway from maternal ACEs to child outcomes.

Mechanisms at the family level make up the ecosystem in which the child develops. Maternal adult attachment insecurity is one such area of interest, given that ACEs may impede the development of secure attachment during childhood, which may have downstream implications for adult romantic relationships. Considerable research has demonstrated that attachment insecurity has been associated with reduced relationship satisfaction (Candel & Turliuc, 2019). Parental negative experiences in romantic relationships (e.g., conflict) may also have implications for reduced mental health, which can subsequently impact parents’ ability to engage in their parenting role (Gartland et al., 2021; Goodman et al., 2011).

### The Current Study

To advance knowledge and inform future directions in the field, as well as prevention and intervention efforts, the current study employed path analysis using a longitudinal cohort study. The final study sample included 1,994 mothers and their children to examine the association between maternal history of ACEs collected when children were age three and child internalizing and externalizing concerns at age eight. Specifically, it was hypothesized that a significant association existed between maternal ACEs and child internalizing and externalizing concerns via the pathways of maternal attachment insecurity, maternal romantic relationship difficulties, and maternal mental health. We did not make specific hypotheses regarding whether these maternal variables would differentially impact child internalizing, compared to externalizing, concerns.

## Method

### Study Design and Participants

Pregnant mothers in the All Our Families (AOF) cohort, an existing longitudinal cohort study of >3,000 Albertan mothers and children (McDonald et al., 2013; Tough et al., 2017), were recruited in pregnancy through primary health care offices, community advertising, and laboratories between 2008-2011. Among those approached, approximately 84% (*n* = 3,387) of mothers agreed to participate; additional details regarding study recruitment have been published elsewhere (McDonald et al., 2013; Tough et al., 2017). Of these families, *n* = 2,909 were eligible based on their child’s age for the data used in the present study, and 69% (*n* = 1,994) completed the questionnaires. Inclusion criteria for all mothers were if: (1) they were ≥18 years of age; (2) able to complete questionnaires written in English; (3) were <24 weeks gestation when recruited; and (4) receiving community-based prenatal care. Babies were born between August 2008 and August 2011. Mothers were asked to complete a survey during their pregnancy and following the birth of their child and again at ages one, two, three, five, and eight years. Data used in the present study consisted of measures completed by the mother when their child was age three, five, and eight. Participant compensation consisted of a gift card for a nominal amount at each timepoint. Study data were collected and managed using Research Electronic Data Capture (REDCap) electronic data capture tools hosted at the University of Calgary. REDCap is a secure, web-based platform designed to support data capture for research studies (Harris et al., 2009, 2019).

The majority of women recruited into this cohort reported household incomes above the median, and were more likely to be married compared to general population samples of women in Calgary, Alberta, and Canada (McDonald et al., 2013; Tough et al., 2017). However, age, education, and likelihood of being born outside of Canada did not differ between women in this sample and those in Calgary and Canada (Tough et al., 2017). Baseline equivalence analyses were conducted to examine any statistically significant differences between the final study sample and the mother-child dyads that dropped out (i.e., did not complete measures). Mothers who completed the study measures had significantly higher educational attainment (*p* < .001), higher income (*p* = .035), were more likely to be White (*p* < .001) and were older (*p* = .002) compared to those who dropped out. Accordingly, maternal age, family income, and maternal educational attainment were controlled for in the present analysis.

### Maternal Measures

#### Maternal ACEs

The ACEs measure included a frequency count of whether an individual had experienced any of the following instances of (scored as either present = 1 or absent = 0): physical, emotional, or sexual abuse; witnessing violence against ones’ mother; or living with household members who experienced problematic substance use, were mentally ill or suicidal, or were ever imprisoned (Felitti et al., 1998). In the current study, maternal exposure to ACEs were retrospectively reported using an adapted 11-item version of the ACEs Checklist when their child was three years of age (Anda et al., 2007; Felitti et al., 1998). The summative ACEs score was coded as a categorical variable (i.e., 0, 1, 2, 3, or 4+ ACEs) in line with previous research (Felitti et al., 1998).

#### Maternal Adult Attachment Insecurity

When the study child was five years of age, maternal attachment insecurity was measured using the Experiences in Close Relationships – Short Form (ECR-S; Wei et al., 2007). This 12-item self-report measure assessed two factors of attachment insecurity, namely avoidance and insecurity. Sample items include “I want to get close to my partner, but I keep pulling back” and “I find that my partner(s) don’t want to get as close as I would like”. All responses are scored on a 7-point Likert scale (1 = “strongly disagree” to 7 = “strongly agree”), where higher total scores reflected greater attachment insecurity. Cronbach’s alphas for attachment avoidance and anxiety were α = .82 and α = .72, respectively.

#### Maternal Anxiety

When the study child was eight years of age, mothers completed the Spielberger’s State Anxiety Inventory – Short form (SSAI-SF; Spielberger et al., 1970). This six-item self-report measure examined current symptoms of anxiety. Response options consisted of a four-point Likert scale (1 = “not at all” to 4 = “very much so”). Higher scores indicated greater symptoms of anxiety and the cut off to indicate clinically significant symptoms was ≥13. Cronbach’s alpha for anxiety symptoms was α = .86.

#### Maternal Depression

When the study child was eight years of age, mothers completed the Center for Epidemiological Studies Depression Scale (CES-D; Radloff, 1977) to assess symptoms of depression occurring in the past week. This measure consisted of 20 items, where higher scores indicated greater depressive symptomology. Responses consisted of a four-point Likert scale (0 = “rarely or none of the time” to 3 = “most or all of the time”) and the cut off score to indicate clinically significant depressive symptomology was >16. Cronbach’s alpha for depression symptoms was α = .91.

#### Maternal Romantic Relationship Functioning

**Figure 1.**
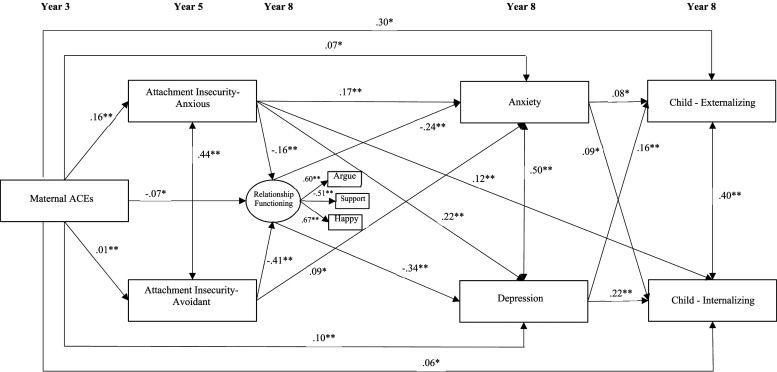
Path analysis examining associations between maternal ACEs and child internalizing and externalizing problems at eight years of age. *p < .05; **p < .01. Only significant effects were reported. Note. Relationship Functioning represents a latent variable consisting of three items: 1) Difficulty working out arguments with partner 2) Self-reported happiness in romantic relationship 3) Satisfaction with socio-emotional support provided by partner.

When the study child was eight years of age, mothers who were in romantic relationships completed several items developed by the original authors assessing various aspects of romantic relationship functioning. Of note, 91.4% (*n* = 1,784) of the sample reported they were married, common-law, or living with a partner at the time of the assessment. A latent variable was created for romantic relationship functioning, consisting of items that examined mothers’ perception of resolving arguments (i.e., 1 = great difficulty, 3 = no difficulty), their satisfaction with perceived socio-emotional support (i.e., 1 = very satisfied, 4 = very unsatisfied), and their overall happiness with their partner (i.e., 1 = extremely unhappy, 7 = perfectly happy) using Likert scale response options (see Figure 1).

### Child Measure

#### Child Internalizing and Externalizing Concerns

When the study child was eight years of age, mothers completed the Behavior Assessment System for Children, Second Edition (BASC-2; Reynolds & Kamphaus, 2004). This 160-item assessment examined mothers’ perceptions of their children and yielded two composite scores, externalizing behaviors (i.e., hyperactivity and aggression) and internalizing behaviors (i.e., depression, anxiety, and somatization). Mothers reported on the frequency of socio-emotional and behavioral problems, ranging from “never” to “almost always”. Cronbach’s alphas for internalizing behaviors and externalizing behaviors were both α = .92.

### Statistical Analysis

Descriptive analyses were completed using IBM SPSS Statistics (Version 29.0.2.0, 2023; IBM Corp, 2023). A mediation framework in context of path analysis was used to test the indirect effects (MacKinnon, 2012). We estimated a multivariate path model to examine the association between maternal ACEs and children’s internalizing and externalizing symptoms via the hypothesized mediators (i.e., attachment insecurity, relationship functioning and maternal mental health). A latent variable was created in Mplus 8.8 (Muthén & Muthén, 1998–2016; see Figure 1), consisting of the three maternal romantic relationship items (i.e., mothers’ perception of resolving arguments, their satisfaction with perceived socio-emotional support, and their overall happiness with their partner). Maternal ACEs were considered the predictor (X), and child internalizing and externalizing were considered the outcomes (Ys). We regressed the mediators onto X, and the Ys were regressed on the mediators, controlling for the direct effects of X on the Ys. Indirect effect sizes were evaluated using completely standardized indirect effects. The indirect effects represent the association between X and Y through the mediators (Kelley & Preacher, 2012). The path analysis was conducted using the maximum likelihood robust estimator, which has demonstrated to be robust to non-normality in existing research (Yuan & Bentler, 2000). Full Information Maximum Likelihood (Graham, 2009) was used to maximize the inclusion of missing data.

## Results

### Model Fit and Sample Descriptives

Descriptive statistics for variables included in the path model are found in Table 1, and bivariate correlations among all variables are presented in Table 2. Model fit was determined (Comparative Fit Index = 0.96, root mean square error of approximation = 0.05). The findings demonstrated that maternal ACEs were directly associated with child internalizing (β = .132, *p* < .001) and externalizing (β = .124, *p* < .001) concerns. Consistent with calibrated guidelines for psychological research, the magnitude of Pearson’s *r* of .10, .20, and .30 were considered small, moderate, and large effect sizes (Funder & Ozer, 2019). Indirect pathways were also observed via maternal attachment insecurity (avoidance and anxiety), maternal relationship functioning, and maternal depressive, but not anxiety symptoms.

**Table 1.** Descriptive Statistics.

Variable	*N* (%)	M	SD
Maternal Race and ethnicity (reported at pregnancy)			
White	2636 (77.8%)		
Asian	380 (11.2%)		
Black/African north American	50 (1.5%)		
Indigenous	32 (0.9%)		
Latinx	78 (2.3%)		
Middle Eastern	58 (1.7%)		
Mixed race/self-identified “other”	118 (3.5%)		
Missing	35 (1%)		
Maternal education (reported at pregnancy)			
Some elementary or high school	3.5%		
Graduated high school	7.3%		
Some college, trade, or university	14.1%		
Graduated college, trade, or university	58.4%		
Some graduate school	2.7%		
Completed graduate school	13%		
Missing	1%		
Annual household income (reported at pregnancy)			
$39,999 or less	8.9%		
$40,000-$79,000	21.2%		
$80,000-$99,000	16.7%		
$100,000 or more	49.3%		
Missing	4%		
Maternal age (reported at pregnancy)		31.16	4.48
Maternal variables			
ACEs score (reported at child 36 months)		1.55	1.75
0 ACEs	747 (37.7%)		
1 ACEs	465 (23.4%)		
2 ACEs	273 (13.8%)		
3 ACEs	205 (10.3%)		
4+ ACEs	294 (14.8%)		
ACEs items			
Live with anyone depressed, mentally ill, or suicidal?	492 (24.7%)		
Live with problem drinker or alcoholic?	362 (18.2%)		
Live with anyone using illegal drugs or abused prescription medications?	165 (8.3%)		
Live with anyone who served prison, jail, or other correctional facility?	59 (3%)		
Parents separated or divorced?	467 (23.5%)		
Parents or adults slap, hit, kick, punch, or beat each other?	302 (15.2%)		
Parent or adult hit, beat, kick, or physically hurt you?	352 (17.7%)		
Parent or adult swear at, insult, or put you down?	722 (36.3%)		
Anyone at least 5 years older or an adult ever touch you sexually?	243 (12.2%)		
Anyone at least 5 years older or an adult try to make you touch them exually?	146 (7.3%)		
Anyone at least 5 years older or an adult force you to have sex?	55 (2.8%)		
ECR anxiety (reported at child 60 months)		16.47	6.39
ECR avoidance (reported at child 60 months)		11.94	5.54
Relationship – Happiness (reported at child 8 years)		4.63	1.56
Relationship – Support (reported at child 8 years)		3.11	0.94
Relationship – Arguments (reported at child 8 years)		1.59	0.57
CES-D depression (reported at child 8 years)		9.22	8.46
CES-D score >16	363 (18.6%)		
SSAI-SF anxiety (reported at child 8 years)		10.14	3.51
SSAI-SF score ≥13	335 (17.4%)		
Child variables			
BASC-2 internalizing concerns T score (reported at child 8 years)		49.06	10.05
BASC-2 externalizing concerns T score (reported at child 8 years)		49.60	8.29

Descriptive statistics for all study variables.

*Note.* ACEs, Adverse Childhood Experiences; ECR-SF, Experiences in Close Relationships-Short Form; SSAI-SF, Spielberger’s State Anxiety Inventory – Short form; CES-D, Center for Epidemiologic Studies Depression Scale; BASC-2, Behavior Assessment System for Children - Second Edition.

**Table 2.** Pearson Correlations Among Study Variables.

	1	2	3	4	5	6	7	8	9	10	11	12	13
1. Maternal age	-												
2. Maternal income	.02	-											
3. Maternal education	.31**	−.02	-										
4. Maternal ACEs	−.005	−.01	−.01	-									
5. ECR- Anxiety	−.03	−.02	.002	.18**	-								
6. ECR- Avoidance	−.001	.01	−.02	.11**	.47**	-							
7. Maternal relationship – Arguments	.01	.003	−.04	.08**	.24**	.34**	-						
8. Maternal relationship – Happiness	.003	.002	.05*	−.10**	−.20**	−.29**	−.39**	-					
9. Maternal relationship – Support	.01	−.04	.04	−.05*	−.18**	−.24**	−.27**	.39**	-				
10. Maternal depression	−.01	.04	−.02	.20**	.40*	.35**	.31**	−.28**	−.25**	-			
11. Maternal anxiety	−.01	.04	−.04	.14**	.30**	.29**	.23**	−.23**	−.16**	.61**	-		
12. Child internalizing	−.06*	−.06*	−.002	−.02	.005	.02	.03	−.01	−.01	−.03	−.01	-	
13. Child externalizing	−.01	−.08**	.02	−.04	.04	.04	.05	−.02	.03	−.01	.01	.46**	-

*Note.* Boldface indicates statistically significant correlations (*p < .05; **p < .01; ***p < .001).

### Path Analysis: Maternal ACEs to Child Internalizing and Externalizing Concerns

The association between maternal ACEs and child internalizing and externalizing concerns at age eight were examined using a path analysis, while controlling for maternal age, maternal education, and household family income. Indirect effects for the association between maternal ACEs and child internalizing and externalizing concerns were examined via maternal attachment insecurity (i.e., anxiety and avoidance), maternal romantic relationship functioning, and maternal mental health (i.e., anxiety and depressive symptoms). Results of the path analysis are presented in Table 3, and the individual paths comprising this model, using standardized scores, are presented in Figure 1.

**Table 3.** Direct and Indirect Effects Linking Maternal ACEs and Child Internalizing or Externalizing Concerns.

Pathway	Direct effect	Total indirect effect	Specific indirect effect
	β [95% CI]	β [95% CI]	β [95% CI]
**ACEs → internalizing**	.132* [.008; .119]	.069* [.048; .089]	
Via attachment anxiety			.018* [.006; .031]
Via depression symptoms			.02* [.008; .037]
Via attachment anxiety → anxiety symptoms			.002* [.000; .005]
Via attachment anxiety → depression symptoms			.008* [.004, .012]
Via relationship → depression symptoms			.006* [.000; .011]
Via attachment anxiety → relationship → depression symptoms			.002* [.001; .003]
Via attachment avoidance → relationship → depression symptoms			.003* [.001; .005]
**ACEs → externalizing**	.124* [.011; .116]	.061* [.041; .081]	
Via depression symptoms			.016* [.005; .028]
Via attachment anxiety → anxiety symptoms			.002* [.000; .004]
Via attachment anxiety → depression symptoms			.006* [.002; .009]
Via attachment anxiety → relationship → depression symptoms			.001* [.000; .002]
Via attachment avoidance → relationship → depression symptoms			.002* [.000; .004]

*Note.* * *p* < .05. For specific indirect effects, we only report those that were significant.

#### Pathways to Internalizing Problems

A significant direct effect was found for the association between maternal ACEs and child internalizing concerns, (β = .06, *p* = .025). Indirect effects (β = .07, *p* < .001) were also found, such that attachment anxiety, relationship functioning, and maternal depression were associated with child internationalizing problems (β = .002, *p* = .006). The pathway from attachment avoidance, relationship functioning, and maternal depression to child internalizing problems was also significant (β = .003, *p* = .005).

#### Pathways to Externalizing Problems

A direct effect was observed from maternal ACEs to child externalizing problems (β = .06, *p* = .018) in addition to indirect effects (β = .06, *p* < .001). Specifically, indirect effects were observed via attachment anxiety, relationship functioning, and maternal depression (β = .001, *p* = .008), and attachment avoidance, relationship functioning, and maternal depression (β = .002, *p* = .010).

Taken together, maternal attachment insecurity, reduced romantic relationship functioning, and maternal depression represent significant mechanisms that account for the association between maternal ACEs and child internalizing and externalizing concerns at age eight. A total of 13.4% of the variance in internalizing problems and 10.3% of the variance in externalizing problems was accounted for by this model.

## Discussion

In this longitudinal study, maternal ACEs were indirectly associated with both child internalizing and externalizing symptoms through several intermediary pathways, including maternal attachment insecurity, romantic relationship functioning, and maternal depression symptoms. Direct pathways were observed between maternal ACEs and both child internalizing and externalizing concerns after including mediating mechanisms, which is consistent with a recent meta-analysis by Racine and colleagues (2023). This study addresses gaps in existing literature by examining multiple psychosocial pathways for the association between maternal ACEs and child internalizing and externalizing concerns. Overall, these results highlight the importance of maternal psychosocial mechanisms that may account for the intergenerational transmission of risk from maternal ACEs to child internalizing and externalizing outcomes.

Attachment theory suggests that early interactions with caregivers lay the foundation for expectations in future relationships. Adult attachment styles have implications for relationships, including romantic relationships (Bartholomew & Shaver, 1998). Thus, maternal ACEs could lead to insecure attachment, both in childhood, and subsequently adulthood, which may have future implications for how individuals interact with others, including their own children. Existing literature suggests that adult attachment insecurity, communication behaviors with relationship partners, and psychopathology interact to perpetuate mental health issues. For example, Kobak and Bosmans (2019) suggested that insecure attachment, characterized by negative expectations that others will not be available or responsive to one’s needs, tend to be associated with interpretative biases. These biases can lead an individual to minimize, ignore, or overlook positive signals from their partners while magnifying perceived threats to the availability of their partner. As a result, these expectations and behaviors create a cycle that sustains perceived threats to the relationship and contribute to mental health concerns. These patterns of behavior may extend to interactions with children, thus possibly increasing risk for mental health concerns in the next generation, highlighting the full cascade of effects from mothers to their children.

Associations observed in the present study can also be interpreted using the Family Stress Model (Conger et al., 2002). Originally conceptualized to describe the impact of economic stress on family functioning, this model has since been extended to elucidate how various family stressors can negatively impact children’s outcomes (Masarik & Conger, 2017). For example, the Family Stress Model posits that the experience of stressors and distress may strain family relationships and negatively impact parenting, ultimately leading to diminished health and well-being among children (Conger et al., 2002; Masarik & Conger, 2017). Thus, it is reasonable to infer that maternal ACEs serve as an initial stressor that persists and initiates a cascade of potential negative outcomes. These outcomes encompass attachment insecurity, reduced quality in romantic relationships, and mental health, which can collectively impede a parent’s ability to engage in parenting. This, in turn, may negatively influence children’s internalizing and externalizing concerns. Overall, exploration of these risk factors was undertaken with the goal of identifying families at highest risk to inform prevention and intervention efforts.

While the present study examined psychosocial mechanisms that may account for the association between maternal ACEs to child internalizing and externalizing concerns, it is important to acknowledge that theoretically, this intergenerational transmission may occur via epigenetic, neurobiological, and physiological pathways (Buss et al., 2017). McLaughlin and colleagues (2014) put forth a conceptual framework suggesting that different types of child adversity may be associated with unique neural outcomes. Past research has supported this dimensional model of adversity, such that early adversity characterized by deprivation, but not threat, was associated with cognitive and emotional functioning outcomes (Carozza et al., 2022). Research has also demonstrated that the association between maternal ACEs and infant physical health was mediated by maternal biomedical risk, including loss of fetal movement during pregnancy, diabetes during pregnancy, and the need for intensive care after birth (Madigan et al., 2017).

### Future Directions

Given that each mediator in this study was measured at a single timepoint, future avenues of research should explore the impact of timing of maternal mental health and relationship functioning on the association between parent ACEs and child outcomes. It is also important to note that several mediators, including maternal relationship functioning and anxiety and depressive symptoms, were measured at the same time point as the child outcomes, thereby limiting the interpretation of results as a mechanism for intergenerational transmission. It is possible that factors such as maternal mental health or relationship functioning have greater impacts for child outcomes during critical periods. For example, past research has highlighted the temporal distinction of prenatal and postnatal depression on child internalizing problems. A study by Hentges et al. (2020) demonstrated that maternal depression at child ages two and three had a stronger association with child internalizing and externalizing symptoms at age five compared to depression prenatally or immediately postpartum (child ages four months and one year; Hentges et al., 2020). Furthermore, the transition to parenthood can be a significant life stressor associated with diminished romantic relationship functioning. This is particularly relevant for parents with insecure attachment styles, who may be at greater risk for decreased well-being in their romantic relationships (Doss & Rhoades, 2017; Rholes et al., 2001).

Additionally, it will be relevant to explore the possibility of a dose–response relationship in the association between maternal mental health concerns and child outcomes. That is, the experience of mental health concerns for extended periods of time during the child’s development may differentially impact child outcomes compared to mothers who experience briefer periods of mental health concerns. Taken together, it is critical to use longitudinal methods with repeated measures to explore the timing and sequence of events and their impacts on child outcomes. This rigorous methodological approach can shed light on critical periods in which families are most vulnerable to these complex dynamics.

While this study highlights several pathways that account for the transmission from maternal ACEs to child internalizing and externalizing outcomes, similar pathways from maternal ACEs to child ACEs have not yet been examined. Research to date has highlighted that parents who were exposed to ACEs, compared to those who were not, were more likely to have children who experienced ACEs (Narayan et al., 2021), which may subsequently increase risk for internalizing and externalizing concerns. There may be several currently untested mediators that account for the association between parent and child ACEs. For example, parental post-traumatic stress disorder may mediate the association between parent and child ACEs (Narayan et al., 2021). Additionally, parent ACEs were associated with increased risk for outcomes such as substance use, intimate partner violence, and mental health concerns, all of which represent ACEs in and of itself (Petruccelli et al., 2019; Zhu et al., 2023). Thus, identification of pathways that could account for transmission of ACEs between generations of the same family could serve to better inform how prevention efforts should be targeted. Future research should also explore potentially adverse or traumatic events that are not captured by the ACEs questionnaire. For example, past research suggests that racism and systemic oppression were significantly associated with mental health concerns (Kirkinis et al., 2021; Vargas et al., 2020). Existing research also suggests that maternal well-being may demonstrate mediation-like effects in the association between economic hardship and ACEs among their children (Liming, 2019). Overall, this highlights that structural inequalities can have far-reaching implications for mothers and children that are not captured by the original ACEs questionnaire.

In future research, it will be critical to examine other facets of romantic relationship functioning beyond those explored in the current study. Existing research suggests that different aspects of romantic relationship functioning may have unique implications for child outcomes. For instance, past research has demonstrated that ACEs were associated with both increased IPV perpetration and victimization (Zhu et al., 2023) which may subsequently have implications for child development outcomes. Gartland and colleagues (2021) demonstrated that child exposure to IPV, which itself represents an ACE, was associated with not only increased mental health concerns, but also sleep problems and impaired language skills among children. Furthermore, it is essential to recognize that, distinct from the romantic relationship is the co-parenting relationship (Margolin et al., 2001). Co-parenting refers to partners acting together in their roles as parents (Feinberg, 2003). A meta-analysis conducted by Teubert and Pinquart (2010) demonstrated that positive co-parenting behaviors, including agreement and cooperation between parents, was associated with fewer child internalizing and externalizing problems. Thus, future research should also focus its attention on strengthening understanding of how different aspects of parental relationships can serve as focal points for prevention strategies aimed at promoting the overall well-being of the family unit.

### Clinical Implications

These findings, as well as a wealth of prior research, highlight the critical need to break cycles of transmission instigated by caregiver ACEs. Prevention and intervention efforts that are trauma-informed and address psychosocial risk factors that could contribute to child mental health concerns are needed. Specifically, trauma-informed care should include experiences that minimize distress, increase patient autonomy, and clinicians should possess an appropriate understanding of trauma symptoms (Reeves, 2015). Additionally, parent skills development for effective parenting and conflict resolution may help foster effective communication and subsequent family well-being. Additionally, psychoeducation that highlights the implications of maternal mental health concerns and parental relationship difficulties on child mental health may be useful as a prevention strategy. For example, past research demonstrated that a mindful parenting intervention was associated with improvement in maternal mental health, parenting self-efficacy and bonding, and mindful parenting (Potharst et al., 2022). Recent research demonstrated that a trauma-informed care approach, marked by standardized screening for childhood trauma and clinician training focused on active listening and empathy, was associated with fewer child adverse birth outcomes among a low-risk maternity clinic (Racine et al., 2021). It is important to acknowledge that mothers with a history of ACEs may experience barriers to accessing intervention and prevention efforts. For example, a history of ACEs may be associated with factors such as low socioeconomic status (Berger et al., 2004), which can interact with mental health concerns and thereby limit the ability to access resources. Thus, incorporation of prevention efforts among mothers may represent a cost-effective way of mitigating adverse outcomes among children, thereby disrupting the intergenerational transmission of risk.

Importantly, these results highlight that the examined pathways do not fully account for negative outcomes in the next generation. The present pathways showed small estimates, indicating that they accounted for a small amount of the variance in child internalizing and externalizing concerns. This suggests the presence of other factors that could mitigate risk for child internalizing and externalizing outcomes among families with maternal ACEs exposure.

Resiliency factors, such as social support, may buffer against the impact of ACEs on maternal, child, and family well-being (Madigan et al., 2016). For example, Narayan and colleagues (2018) demonstrated that benevolent childhood experiences, including the presence of a supportive caregiver, friend, or predictable routines, predicted lower stressful life events and PTSD symptoms, beyond the effects of ACEs. As such, social support may represent an important and modifiable risk factor that can mitigate the cascade of risk following ACEs exposure. Further, this research exclusively examined factors within the family, however children, particularly during middle childhood, are exposed to other social environments, including school and extracurricular activities that could represent important sources of social support and influence. Overall, this highlights the importance of using the present results to contextualize and inform methods for building strengths and resiliency among families at elevated risk for adverse outcomes following ACEs exposure.

### Study Limitations

Several limitations of the current study are worthy of note. To begin, this study was limited by the measurement of maternal relationship functioning, which was assessed using three items that were not drawn from a standardized questionnaire. Of note, all other questionnaires included this study were standardized, and this present choice may have been made in an attempt to reduce participant burden. However, use of these items may have negatively impacted validity and reduced generalizability to past and future research examining relationship functioning due to use of different measures. Thus, future research should explore facets of romantic relationship functioning in a manner that is consistent with established measures. Additionally, all measurements included in the study were single informant, with responses provided by the child’s mother. Given that the use of multi-informant assessments may provide valuable contributions to understanding children’s internalizing and externalizing concerns (Renk, 2005), the use of single-informant measures represents a limitation of the present study. This study utilized a count score of ACEs, and while significant research has demonstrated a dose-response effect in the association between number of ACEs experienced and severity of health outcomes across various domains, use of a count score does not provide contextual information regarding the types of adversity experienced. This may be particularly relevant given that different types of adversity, such as those characterized by deprivation (e.g., neglect) and threat (e.g., abuse) may have unique impacts on development (McLaughlin et al., 2014).

Secondly, the sample that this research was drawn from, which consisted of primarily White, educated, and middle to upper-middle class mothers, represented a limitation. Over 77% of mothers in this sample self-identified as White, however, it is important to note that this demographic breakdown is generally consistent with the geographical location the sample was drawn from.

Thirdly, it is important to note that the present study was unable to examine the association between parent ACEs and child mental health outcomes among fathers. This limitation arises from the fact that our sample was drawn from a larger pregnancy cohort that consisted solely of self-identified mothers. It is worth emphasizing that despite fathers playing an increasingly important role in parenting responsibilities, fathers remain understudied in child development research (Cowan & Cowan, 2019). Thus, future research should be dedicated to understanding the pathways connecting parent ACEs to child outcomes, accounting for both parents. Additionally, understanding how the attachment style of one parent may influence the other, and relatedly, the implications for child outcomes, should be a central focus in future investigations to inform how complex family dynamics may interact to influence child well-being.

### Conclusion

This study demonstrated that retrospectively recalled maternal ACEs were associated with child internalizing and externalizing concerns at age eight via psychosocial pathways including maternal attachment insecurity, maternal romantic relationship functioning, and maternal mental health. The findings suggest that parenting prevention and intervention efforts should adopt a skill development and/or trauma-informed approach and focus on fostering resiliency factors that may mitigate the downstream consequences of ACEs exposure in subsequent generations.
